# Waterborne Viruses: A Barrier to Safe Drinking Water

**DOI:** 10.1371/journal.ppat.1004867

**Published:** 2015-06-25

**Authors:** Aimee M. Gall, Benito J. Mariñas, Yi Lu, Joanna L. Shisler

**Affiliations:** 1 Department of Civil and Environmental Engineering, University of Illinois at Urbana-Champaign, Urbana, Illinois, United States of America; 2 Safe Global Water Institute, University of Illinois at Urbana-Champaign, Urbana, Illinois, United States of America; 3 Department of Chemistry, University of Illinois at Urbana-Champaign, Urbana, Illinois, United States of America; 4 Department of Microbiology, University of Illinois at Urbana-Champaign, Urbana, Illinois, United States of America; 5 College of Medicine, University of Illinois at Urbana-Champaign, Urbana, Illinois, United States of America; University of Michigan Medical School, UNITED STATES

## What is the Global Status of Access to Safe, Pathogen-Free Drinking Water?

Nearly 25% of the global population (1.8 billion people in 2012) is consuming fecally-contaminated water [1]. This water can contain bacteria, protozoa, and viruses that can cause a variety of diseases in humans, most notably gastroenteritis. The impact on public health is staggering. Unsafe water, inadequate sanitation, and poor hygiene are responsible for about 90% of diarrheal deaths worldwide [2]. Not surprisingly, diarrhea is the second leading cause of death for children under the age of five globally (1.2 million deaths in 2012) [2]. In addition to the human cost, the World Bank estimates that lack of access to safe water and sanitation results in a global economic loss of US$260 billion annually [3].

The lack of access to improved water disproportionally affects those living in poverty in rural, developing regions; however, even populations living in countries with state-of-the-art water and waste treatment facilities are prone to waterborne disease outbreaks. For example, there were at least 33 outbreaks associated with drinking water reported in the United States of America during 2009–2010 [4]. Regardless of the socioeconomic status of a country, illnesses due to contaminated drinking water are considered significantly underreported because people do not seek medical attention for self-limiting infections and because of the current limitations on clinical detection of virus infections [4].

## Are Waterborne Viruses a Particular Concern?

It is well known that bacteria are major causes of diarrhea transmitted through unsafe drinking water. What is less appreciated are viruses in these same drinking water sources and their impact on human health. Water-transmitted viral pathogens that are classified as having a moderate to high health significance by the World Health Organization (WHO) include adenovirus, astrovirus, hepatitis A and E viruses, rotavirus, norovirus and other caliciviruses, and enteroviruses, including coxsackieviruses and polioviruses [5]. Also, viruses that are excreted through urine like polyomaviruses [5] and cytomegalovirus [6] can potentially be spread through water. Other viruses, such as influenza and coronaviruses, have been suggested as organisms that can be transmitted through drinking water, but evidence is inconclusive [5].

Most of the above viruses are most commonly associated with gastroenteritis, which can cause diarrhea as well as other symptoms including abdominal cramping, vomiting, and fever. It should be noted that some of these same viruses could also cause more severe illnesses including encephalitis, meningitis, myocarditis (enteroviruses), cancer (polyomavirus), and hepatitis (hepatitis A and E viruses) [5]. Hepatitis E virus can also cause a mortality rate of up to 25% in pregnant women [5]. Viral infections are usually self-limiting in healthy individuals. They can cause greater morbidity in children under the age of five, the elderly, immunocompromised people, and pregnant women. Waterborne virus-based diseases may be higher in developing regions, where there is widespread malnutrition and large populations of HIV-positive people. Regardless, there are few broad spectrum anti-viral drugs to treat these diseases.

Certainly, there is good cause for controlling these waterborne viruses. Rotavirus, for example, is the leading cause of severe acute diarrhea in children under the age of five globally, resulting in over half a million deaths annually [7]. The worldwide use of new rotavirus vaccines may make the removal of this virus from water supplies less of an issue in the future. Similarly, better coverage with vaccinations against hepatitis A and poliovirus would also greatly decrease the health risks of these viruses in drinking water.

Waterborne viruses differ in terms of their genome content and capsid proteins, but these viruses share several properties that make them of particular concern regarding the risk of disease outbreak associated with drinking water contamination. Several of these viruses have extremely low infectious doses; the probability of infection from exposure to one rotavirus particle is 31% [8]. Viruses are shed in feces in very high numbers even asymptomatically. For example, up to 10^11^ norovirus particles can be present per gram of stool [9]. In addition, non-enveloped viruses can persist in water for long periods of time [10]. When considering these characteristics, inadequate disinfection of fecally contaminated drinking water could easily lead to outbreaks of viral gastroenteritis from ingestion. Notably, drinking water can also transmit viruses via inhalation (e.g., showering) or contact with skin and eyes (e.g., swimming) causing respiratory and ocular infections.

## What is the State of the Art for Control of Viruses in Water?

Water treatment utilities routinely assay for the presence of fecal coliforms in water supplies, but they do not assay for the presence of infectious viruses because it is either impossible or not feasible to detect or propagate infectious virus particles in a cost-efficient and timely manner. Despite these barriers, the United States Environmental Protection Agency (USEPA) is evaluating adenovirus, caliciviruses, enteroviruses, and hepatitis A virus for potential regulatory action [11]. The current US regulations require the removal or inactivation of 99.99% of enteric viruses by approved treatment techniques, but specific virus families are not individually regulated. These approved treatment techniques are based on bench scale studies where a specific virus is exposed to a disinfectant at various environmental conditions until reaching 99.99% inactivation. By studying a range of enteric viruses, regulations are decided for each disinfectant based on an appropriate dose to adequately inactivate the most resistant enteric virus studied. Utilities must apply an appropriate disinfectant dose to meet enteric virus regulations.

Common water treatment techniques worldwide include physically removing pathogens through conventional treatment and inactivating pathogens by applying ultraviolet light or chemical oxidants such as chlorine, chloramines, ozone, and chlorine dioxide. Since viruses are so small, conventional treatment, including filtration, is ineffective at physically removing viruses. The application of disinfectants highly depends on water chemistry and local regulations. Free chlorine (i.e., sum of hypochlorous acid and hypochlorite ion formed by dissolution and hydrolysis of chlorine gas in water) is the most commonly used disinfectant worldwide and has been used to disinfect water since the early 1900s [12]. Most viruses are inactivated by this strong oxidant. However, free chlorine treatment may produce regulated toxic disinfection by-products (DBPs), and it is ineffective to control *Cryptosporidium*, a protozoan that is transmitted in water and causes diarrhea [13,14]. Thus, some drinking water utilities are moving towards using monochloramine (i.e., formed by mixing chlorine and ammonia with the latter in slight excess) to control the formation of regulated toxic DBPs, and either monochromatic (~254 nm) or polychromatic (200–300 nm) ultraviolet (UV) light for controlling both DBP formation and *Cryptosporidium* contamination. Unfortunately, these changes in disinfection practice come at a cost to virus control. For example, while adenovirus is susceptible to inactivation by free chlorine, it is highly resistant to inactivation by both monochloramine and UV light [15]. Chlorine dioxide and ozone, also strong oxidants, are both effective at controlling viruses, but they have operational challenges, such as the need for on-site generation, and the production of DBPs, including chlorite from chlorine dioxide and bromate from ozone [12].

Regardless of the disinfectant applied at a drinking water utility, as the treated water travels from the treatment plant to the tap, cross-contamination can occur throughout the miles of water distribution infrastructure due to cavitation and accidental depressurization; therefore, the use of secondary disinfectants in distribution systems is required. Unfortunately, the only two disinfectants capable of maintaining a residual in the distribution system are free chlorine and monochloramine. Although free chlorine is a stronger disinfectant with respect to pathogen inactivation, monochloramine provides a more stable residual in distribution systems, and so both are utilized.

## What are the Barriers toward Disinfecting Viruses in Drinking Water?

There are several barriers that prevent viruses in drinking water from being detected. From a technological standpoint, as compared to detecting fecal coliforms, virus propagation requires the use of tissue culture, a system that requires increased time, labor, expertise, and expensive equipment [8]. Furthermore, several of these viruses cannot be grown easily (adenovirus serotypes 40 and 41) or at all (human norovirus, hepatitis A virus) in cell culture. Consequently, traditional viral growth assays (plaque assays) are either unavailable or too lengthy in time to be practical for water treatment facilities. For example, a 10-day incubation period is required to detect replicating adenoviruses via plaque assays. While ELISA or qPCR-based technologies can be used to rapidly detect viral proteins or genomes, respectively, they do not distinguish infectious versus non-infectious viral particles. Integrated cell culture-PCR (ICC-PCR) reduces time requirements of traditional plaque assays and allows for infectious viruses to replicate in host cells, but it still employs the use of cell culture that is impractical at water treatment utilities [8]. Although there have been advances in concentrating viruses from large volumes of water [16], there has yet to be a rapid way to detect viable viruses.

In addition to these detection technology limitations, there is not one “silver bullet” water treatment that will inactivate all virus types independently of water quality. For example, human adenovirus is nearly five times more resistant to monochromatic (254 nm) UV inactivation compared to other enteric viruses [17]. While bacteriophages are often used as models to study enteric eukaryotic viruses, no bacteriophage studied to date accurately represents enteric virus behavior for all disinfectants. The scientific community does not yet understand why viruses have different profiles of resistance to different disinfectants. Regardless, from a regulatory standpoint, a major barrier is that not one disinfection method is effective against all viruses that can be applied to all water quality conditions.

Waterborne viruses have a range of genome types (e.g., DNA, RNA, linear, segmented) and capsid protein structures that contribute to their resistance or susceptibility to specific disinfectants. Many studies have determined reaction rates of disinfectants with amino acids and nucleotides, and this information can be useful in analyzing the mechanism of virus inactivation. However, these data are not always predictive due to the complex nature of viral capsid structures and secondary reactions [18]. It was initially thought that UV treatment of viruses would damage the viral genome as the mechanism for disinfection, whereas chemical oxidants like free chlorine would damage viral capsid proteins as a means for disinfection. UV light is known to be more reactive with nucleotides than amino acids; however, UV light can also damage viral proteins [19], suggesting that UV light has multiple mechanisms to disinfect viruses. For example, UV irradiation inactivates bacteriophage MS2 by both site-specific backbone cleavage of the major capsid protein resulting in genome injection inhibition and by damaging the RNA genome leading to genomic replication inhibition [19]. Whether this is true for other viruses is unknown. Chemical oxidants like free chlorine typically have higher reaction rates with amino acids, but have been shown to damage both viral proteins and genomes [19–21]. Regardless, determining precisely how a disinfectant damages and neutralizes specific viruses is necessary to fully understand the mechanisms of virus inactivation.

## What is the Future of Waterborne Virus Research?

**Fig 1:**
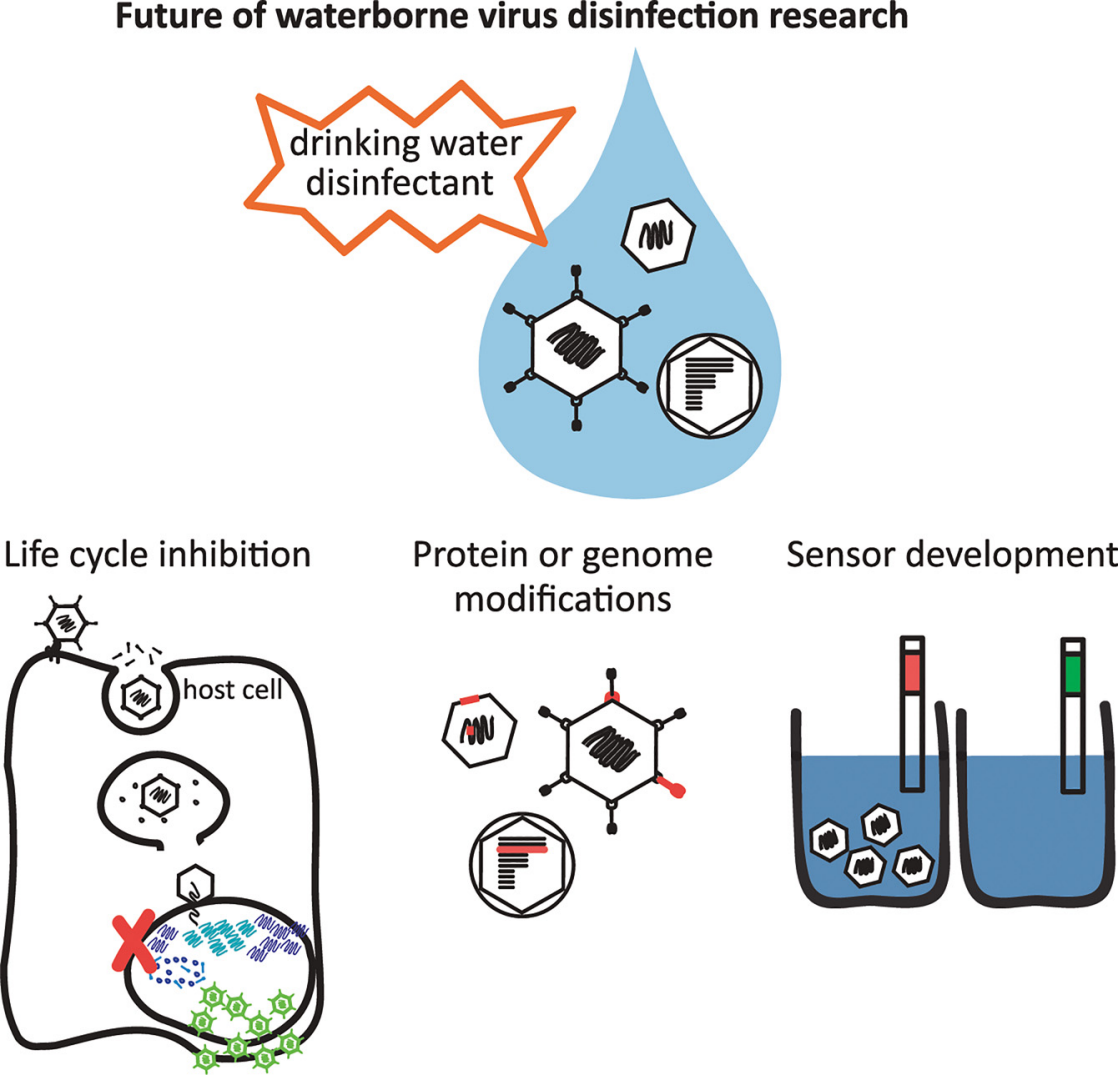
Future of waterborne virus research to provide safe drinking water globally. Gaining a better understanding of how viruses become inactivated by disinfectants requires detailed studies of many virus types and disinfectants to determine what stage of the virus replication cycle becomes blocked, and what modifications to the viral protein and/or genome lead to inactivation. The development of sensors to detect infectious viruses in drinking water will benefit from these studies and is also necessary to ensure safe drinking water.

How does the scientific community overcome the technological and knowledge barriers? First, we must increase our fundamental understanding of how individual viruses become inactivated by disinfectants on a molecular level. This will require detailed studies of the individual components of a viral particle as well as the virion as a whole (Fig 1). The state-of-the-art technique is to correlate virus disinfection with a block in the virus replication cycle as a means to pinpoint what alteration of the protein capsid and/or the viral genome by the disinfectant results in loss of infectivity [19,22]. Technologies including mass spectrometry will afford the opportunity to understand, on an amino acid level, how a disinfectant modifies viral capsid proteins, and how this may result in a non-infectious virus particle [18]. Labeling techniques can also be utilized to determine if viral capsid proteins have undergone specific modifications, such as using a biotin hydrazide that forms covalent bonds with carbonyl groups [23], one of many oxidative products formed on amino acids. The scientific field is now beginning to capitalize on the sensitivity of qPCR or RT-qPCR to detect specific regions of a viral genome that are damaged by a disinfectant. Disinfectant-induced genomic damage can block viral DNA/RNA replication in host cells or viral genome amplification in PCR reactions [24]. However, PCR cannot elucidate what type of genome modification is caused by a disinfectant (e.g., pyrimidine dimers, crosslinking to proteins, chlorine-carbon bonds) or if the host cell is able to repair the lesions [17,24]. A variety of techniques will need to be used and developed to determine on a molecular level how a disinfectant neutralizes a virus and what stage of the replication cycle is blocked. This will likely vary for different viruses and for each disinfectant.

A grand challenge remains to design technology to rapidly distinguish infectious from damaged or portions of viral particles. Recently developed methods combine the antibody-based capture of viral capsids and subsequent qPCR amplification of genomes as a strategy to quantify structurally intact viruses in environmental samples [25]. This immunocapture-qPCR (IC-qPCR) technique is promising to detect infectious viral particles rather than fragments of viral nucleic acid and proteins using ELISA- or qPCR-based techniques alone. Another recent technique employs dyes (ethidium monoazide and propidium monoazide) that can penetrate damaged viral capsids and intercalate into viral nucleic acids inhibiting PCR amplification [26]. This method, like IC-qPCR, requires an intact viral capsid to detect an infectious virus. Unlike IC-qPCR, these dye-based assays rely on the mechanism of inactivation to be a leaky capsid structure. Results from these assays must be interpreted carefully because it is possible that a disinfectant modifies a capsid protein responsible for a key replication cycle event, like attachment, but does not affect the capsid structure or genome. Another new, promising technology to meet this grand challenge relies on the use of aptamers to selectively detect infectious virus particles. For this to occur, there is an in vitro selection from a large library of nucleic acids of aptamers that will selectively bind surface receptors of infectious viruses, but not those from non-infectious viral particles [27]. This technology has already been used in sensors to detect toxins and metals, and many aptamers have been selected for viral proteins and whole viruses for use in antiviral agents [28]. Indeed, an aptamer-based sensor can already distinguish between viable and heat-inactivated vaccinia virus, showing the promise of this technology for detecting other viruses [29]. Because aptamers are much more stable and cheaper than antibodies used in ELISA, such a technology would allow rapid detection of infectious viruses more cost-effectively and selectively.

Once we understand how disinfectants inactivate viruses, we can develop effective treatment protocols for water utilities and the next generations of sensors that rapidly detect and quantify infectious viruses in finished drinking water. Populations most vulnerable to unsafe drinking water live in rural and periurban areas of developing countries. Thus, scientists and engineers must design protocols and sensors to be cost-effective, rugged, and easy to use for these populations. Ultimately, the fundamental knowledge of how viruses become inactivated will ensure better control of viruses in drinking water, increasing access to safer drinking water globally.
